# Multiple Visual Rating Scales Based on Structural MRI and a Novel Prediction Model Combining Visual Rating Scales and Age Stratification in the Diagnosis of Alzheimer's Disease in the Chinese Population

**DOI:** 10.3389/fneur.2019.00093

**Published:** 2019-02-20

**Authors:** Zhenhua Yuan, Chuzheng Pan, Tingting Xiao, Menghui Liu, Weiwei Zhang, Bin Jiao, Xinxiang Yan, Beisha Tang, Lu Shen

**Affiliations:** ^1^Department of Neurology, Xiangya Hospital, Central South University, Changsha, China; ^2^Department of Radiology, Xiangya Hospital, Central South University, Changsha, China; ^3^National Clinical Research Center for Geriatric Disorders, Central South University, Changsha, China; ^4^Key Laboratory of Hunan Province in Neurodegenerative Disorders, Central South University, Changsha, China; ^5^Parkinson's Disease Center of Beijing Institute for Brain Disorders, Beijing, China; ^6^Collaborative Innovation Center for Brain Science, Shanghai, China; ^7^Collaborative Innovation Center for Genetics and Development, Shanghai, China; ^8^Key Laboratory of Organ Injury Aging and Regenerative Medicine of Hunan Province, Changsha, China

**Keywords:** visual rating scales, Alzheimer's disease, sensitivity, specificity, Chinese population, prediction model

## Abstract

**Objective:** To explore the value of multiple visual rating scales based on structural MRI in the diagnosis of Alzheimer's disease (AD) in the Chinese population.

**Materials and Methods:** One hundred patients with AD and 100 age- and gender- matched cognitively normal controls were enrolled in this study. All the participants underwent neuropsychological tests and a structural MRI scan of the brain, among them, 42 AD cases and 47 cognitively normal controls also underwent 3D-T1 weighted sequence used for the analysis of voxel-based morphometry (VBM). The AD cases were divided into mild and moderate–severe groups according to the mini-mental state examination. Each participant was evaluated by two trained radiologists who were blind to the clinical information, according to the six visual rating scales, including for medial temporal lobe atrophy (MTA), posterior atrophy (PA), anterior temporal (AT), orbitofrontal (OF) cortex, anterior cingulate (AC), and fronto-insula (FI). Finally, we estimated the relationship between the visual rating scales and the volume of corresponding brain regions, using correlation analysis, and evaluated the specificity and sensitivity of every single scale and combination of multiple scales in the diagnosis of AD, using a receiver operating characteristic (ROC) curve and establishing a logistic regression model.

**Results:** The optimal cutoff of all six visual rating scales for distinguishing AD cases from normal controls was 1.5. Using automated classification based on all six rating scales, the accuracy for distinguishing AD cases from healthy controls ranged from 0.68 to 0.80 (for mild AD) and 0.77–0.90 (for moderate–severe AD), respectively. A diagnostic prediction model with a combination of MTA and OF results was made as follows: Score = B_MTA(score)_ + B_OF(score)_ −1.58 (age < 65 years); Score = B_MTA(score)_ + B_OF(score)_ −4.09 (age ≥65 years). The model was superior to any single visual rating scale in the diagnosis of mild AD (*P* < 0.05).

**Conclusion:** Each of the six visual rating scales could be applied to the diagnosis of moderate-severe AD alone in the Chinese population. A prediction model of the combined usage of MTA, OF, and age stratification for the early diagnosis of AD was preliminarily established.

## Introduction

Alzheimer's disease is a neurodegenerative disorder mainly characterized by an insidious but progressive loss of memory, accompanied by personality changes and behavior disorders. It is the most common type of dementia in the elderly, and the prevalence is 11% in those over the age of 65 years, and as high as 32% in those over the age of 85 years (1). Early diagnosis of AD is of great importance to the treatment, management, and prognosis (2, 3). Molecular biomarkers contributing to the diagnosis of AD are becoming available but are not widely used in clinical practice. As a common screening means of AD, structural magnetic resonance imaging (MRI) plays a key role in the diagnosis of AD and has been included in the diagnostic guidelines (4–6). Although a number of sophisticated analysis methods are available to quantify global and regional atrophy from MRI, visual rating scales are highly efficient, rapid, and practical tools in clinical practice.

At the earliest, Scheltens et al. put forward a visual rating scale used to evaluate medial temporal lobe atrophy (MTA) in 1992 (7). The sensitivity and specificity of MTA were 81 and 67%, respectively, so it was considered one of the image markers of AD. In many studies, the rating scale (MTA) was subsequently applied. Recently, it has been included into the diagnostic guidelines for AD (4, 6, 8). In 2011, Koedam et al. put forward another evaluation method, posterior atrophy (PA), focusing on the structural changes of the posterior cingulate sulcus, precuneus, parieto-occipital sulcus, and the parietal cortex (9). The sensitivity and specificity were 58 and 95%, respectively. In addition, several other visual rating scales including anterior temporal (AT), orbitofrontal cortex (OF), anterior cingulate (AC) and fronto-insula (FI) were consecutively put forward (10–14). Recently, through evaluation of T1-weighted imaging in 184 post-mortem confirmed dementia patients, Harper et al. found that the combination of six visual rating scales was better than any single rating scale in the diagnosis and differential diagnosis of AD (15). The sensitivity and specificity of the established equation based on six visual rating scales were 94 and 89%, respectively, in distinguishing AD patients from normal controls.

However, no study has stated the value of multiple visual rating scales in the diagnosis of AD in China. To address this gap, we conducted a study to explore the value of multiple visual rating scales based on structural MRI in the diagnosis of AD in the Chinese population and to combine the aforementioned visual rating scales to establish a simple and effective prediction model for early diagnosis of AD.

## Materials and Methods

### Subjects

This study included 102 AD cases (mild AD: 43, moderate and severe AD: 59). All the patients were enrolled in the Department of Neurology of Xiangya Hospital of Central South University, either in the outpatient or inpatient setting between May 2015 and April 2017. The study also included 101 age-matched, gender-matched and cognitively normal controls from the Health Management Center of Xiangya Hospital. This study was approved by the Ethics Committee of Xiangya Hospital of Central South University in China (equivalent to an Institutional Review Board) and was carried out in accordance with the approved guidelines and regulations. Written informed consent was obtained from each participant.

**Inclusion criteria of AD cases:**

1. All the patients should meet the diagnostic criteria of “clinical probable AD” established by the National Institute of Neurological and Communicative Disorders and Stroke and the Alzheimer's Disease and Related Disorders Association (NINCDS-ADRDA) in 2007 (4).
2. All the patients underwent the neuropsychological tests, including the Mini-Mental State Examination (MMSE), Activity of Daily Living (ADL) scale, Clinical Dementia Rating (CDR) and Neuropsychiatric Inventory (NPI).
3. Each participant underwent a structural imaging scan of brain through 3.0 Tesla MRI (Signa HDX, General Electric Healthcare, Milwaukee, WI, United States).

**Exclusion criteria of AD cases:**

1. Patients with dementia caused by cerebral vascular diseases, poisoning, central nervous system infection, anemia, trauma, and other diseases and patients with other degenerative dementias (frontotemporal dementia, dementia with Lewy body and so on).
2. The patients who could not complete the MRI scan due to embedded metal objects in the body (dentures, stents, pacemaker, metal fixtures).
3. Pregnant and lactating women.
4. Subjects with a severe systemic disease (patients with severe hepatic disease, or a long history of chronic hepatic disease and the alanine aminotransaminase (ALT) and aspartate aminotransaminase (AST) exceed the 1.5 times the upper limit; patients with renal dysfunction; with uncontrolled hypertension; with uncontrolled hyperglycemia; with severe cardiac, pulmonary, or hematological diseases).

**Inclusion criteria for cognitively normal controls:**

1. No complaint of cognitive impairment.
2. The score of the MMSE test was in the normal range.
3. Age, sex, and the years of education should be matched with the AD cases.

**Exclusion criteria of cognitively normal controls:**

1. Subjects who could not complete the MRI scan due to embedded metal objects in the body (dentures, stents, pacemaker, metal fixtures).
2. Pregnant and lactating women.
3. Subjects with a severe systemic diseases (patients with severe hepatic disease, or a long history of chronic hepatic disease and the alanine aminotransaminase (ALT) and aspartate aminotransaminase (AST) exceed the 1.5 times the upper limit; patients with renal dysfunction; with uncontrolled hypertension; with uncontrolled hyperglycemia; with severe cardiac, pulmonary, or hematological diseases).

### MRI Scanning

Brain MR imaging was performed on a 3.0 T MRI scanner (Signa EXCITE, General Electric, Fairfield, Connecticut, United States) at Xiangya Hospital Imaging Center. For all MRI procedures, the head was immobilized using self-expanding foam cushions. Volumetric (3D) T1-weighted images were acquired: with thickness/gap = 3/1 mm, echo time (TE) = 30 ms, pulse repetition time (TR) = 2,000 ms, TI = 380 ms, flip angle = 90^?^, matrix = 64 × 64, field of view (FOV) = 220 × 220 mm, voxel size = 0.5 × 0.5 × 0.5 mm. All the participants underwent structural MRI brain scanning (including T1 weighted sequence, T2 weighted sequence, T2 FLAIR sequence and T1 weighted coronal thin layer scanning), among them, 42 AD cases and 47 normal controls also underwent 3D-T1 weighted sequence used for the analysis of voxel-based morphometry (VBM).

### Data Collection of Visual Rating Scales Evaluation and Voxel-Based Morphometry (VBM)

**Figure 1:**
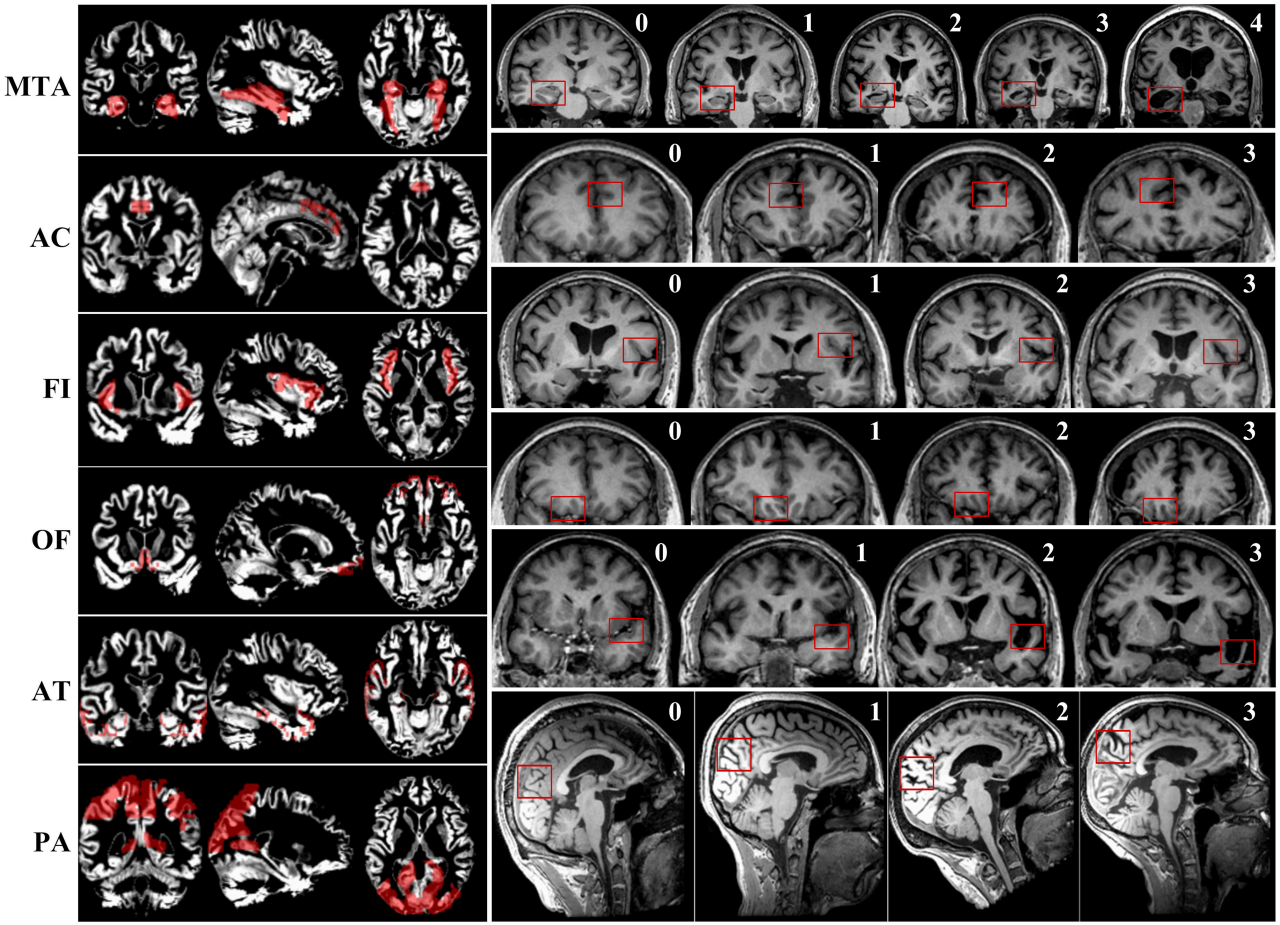
The maps of gray matter signal of corresponding brain regions and examples of scoring of six visual rating scales.

First, two radiologists with at least 10 years of working experience in neuroimaging were trained on consistency of visual rating scales evaluation. Subsequently, visual rating of the T1 weighted sequence of all included participants was performed by the two trained radiologists blind to all clinical and pathological information. Six brain regions were rated according to existing scales. Detailed evaluating rules of the six visual rating scales were described in the previous studies (7, 9–14) and Figure 1. To improve the consistency of rating, two selected radiologists were trained several times, and slice selection of the structural MRI was specified.

To explore the relationship between each rating scale and pattern of gray matter volume loss, VBM was performed using SPM-8 (Statistical Parametric Mapping, Version8) and MATLAB2010a (uk.mathworks.com/products/matlab). In total, 89 individuals, including 42 AD cases and 47 normal controls, were enrolled to perform the analysis of VBM. Because the preprocessing and analysis of original images varied with different sequences, we needed to classify the original images before processing the data from the MRI. In this study, Dcm2AsiszImg software was used to complete the classification of original images. Before statistical analysis, the classified data should have undergone specified preprocessing achieved by using MATLAB2010a and SPM-8. The processing flow of images included motion correction, spatial normalization and segmentation and smoothness of brain tissue imaging. The realignment of head movement aimed to reduce the impact of noise produced by head movements on signal. We used the EPI template of SPM8 to normalize the image data of all subjects and transformed the original images into template images in units of a volume of 3 × 3 × 3 mm. Subsequently, correction for local nonlinear deformation was performed to eliminate local subtle difference and register the data of all subjects into the Montreal Neurological Institute (MNI) space. Gray matter, white matter, and cerebrospinal fluid (CSF) maps were obtained using the unified segmentation approach (16). We used 4 × 4 × 4 full-width half-maximum (FWHM) function to smooth the space to reduce spatial signal-to-noise ratio further and the error caused by space normalization to individuals.

We selected the corresponding brain regions of MTA, PA, OF, AC, AT, and FI, then used Representational State Transfer (REST) software to make a mask. Eventually, the mask was used to extract the gray matter signal of corresponding brain regions. The maps of gray matter signal of corresponding brain regions are presented in Figure 1.

### Consistency Evaluation

#### The Assessment of Consistency Between Raters

Intra-class correlation (ICC) is one of the reliability coefficients to evaluate the interobserver reliability and test–retest reliability. The value of ICC ranges from 0 to 1. A value of ICC lower than 0.4 indicates poor reliability, a range from 0.4 to 0.75 indicates ordinary reliability, and higher than 0.75 indicates good reliability. It is generally acknowledged that the value of ICC should be higher than 0.70 (17, 18).

#### Correlation Analysis Between the Score of Each Visual Rating Scale and Gray Matter Signal of Corresponding Brain Region

To ascertain whether the visual rating scales can really reflect the atrophy of corresponding brain regions, we took the intracranial volume, age, and gender as control variables, and performed partial correlation analysis to estimate the correlation between the score of each visual rating scale and the gray matter signal of the corresponding brain region.

### Exploration of the Value of a Single Visual Rating Scale in the Diagnosis of AD

According to the evaluation results of each visual rating scale, the receiver operating characteristic (ROC) curve was drawn to ascertain the optimal cutoff to diagnose AD, and the sensitivity, specificity, and area under the ROC (AUC) of each visual rating scale were calculated respectively.

### Exploration of the Value of Combining Multiple Visual Rating Scales in the Early Diagnosis of Alzheimer's Disease (Establishment of the Logistic Regression Equation)

The individuals were divided into two groups according to age (age ≥65 years; age < 65 years). The scores (rounded to the nearest integer) of the six visual rating scales and age were enrolled as the concomitant variables of the regression equation. Given that the preceding variables were ordered multivariate statistics, each visual rating scale was set as a dummy variate and diagnosis was set as the dependent variate. A model was established through stepwise selection of the binary logistic regressions. Finally, the optimal model that could distinguish the mild AD cases from the normal controls was ascertained according to the variation of −2 log likelihood.

### Statistical Analysis

All data processing and analyses were performed using SPSS v.21.0 (IBM, West Grove, Pennsylvania, USA) software. Measurement data were presented as means and standard deviations (SDs), and categorical data were presented as proportions. Differences of the measurement variables were tested using two-sample Student's *t*-test or analysis of variance test. Differences of the categorical variables were tested by the 2 tests. Differences of the ranked data were compared using the Wilcoxon rank sum test. The correlation analysis of the two groups of measurement data was performed using partial correlation analysis. The correlation analysis of categorical variables was performed using logistic regression analysis. For all statistical tests, *p* < 0.05 was considered significant.

## Results

### Demographic and Clinical Data

In the early quality control of the imaging data sets, three subjects (2 patients and 1 normal control) were excluded due to excessive head movements during the MRI scan. Ultimately, a total of 200 subjects were enrolled in this study, including 100 patients meeting the diagnostic criteria of “clinical probable AD” and 100 age- and gender- matched healthy controls. The demographic and clinical data of the enrolled subjects are described in Table 1.

**Table 1:** Demographic and clinical data of the AD cases and normal controls.

	**AD cases**	**Normal controls**	***p*-value**	**Cohen'd**
Number	100	100		
Age (years)	63.44 ± 9.97	63.46 ± 8.17	0.99^a^	
Gender (M/F)	31/69	35/65	0.65^b^	
MMSE	15.70 ± 6.60	27.50 ± 1.30	−	
Education (years)	9.73 ± 2.76	9.07 ± 2.90	0.72^a^	
CDR	1.47 ± 0.61	−	−	
ADL	28.13 ± 8.4	−	−	
NPI	10.78 ± 13.1	−	−	
MTA_L	2.32 ± 1.05	1.03 ± 0.54	< 0.01^c^	1.545
MTA_R	2.45 ± 1.06	1.04 ± 0.62	< 0.01^c^	1.624
PA_L	2.21 ± 0.81	1.31 ± 0.63	< 0.01^c^	1.240
PA_R	2.21 ± 0.81	1.31 ± 0.63	< 0.01^c^	1.240
AT_L	2.01 ± 0.73	1.13 ± 0.64	< 0.01^c^	1.282
AT_R	2.00 ± 0.70	1.06 ± 0.53	< 0.01^c^	1.514
OF_L	1.96 ± 0.74	0.90 ± 0.44	< 0.01^c^	1.741
OF_R	1.96 ± 0.76	0.88 ± 0.47	< 0.01^c^	1.709
AC_L	1.87 ± 0.77	0.85 ± 0.48	< 0.01^c^	1.590
AC_R	1.90 ± 0.77	0.84 ± 0.49	< 0.01^c^	1.642
FI_L	2.11 ± 0.77	1.17 ± 0.57	< 0.01^c^	1.456
FI_R	2.11 ± 0.79	1.16 ± 0.56	< 0.01^c^	1.449

a *p-value was calculated by student's t-test*.

b *p-value was calculated by χ^2^-tests*.

c *p-value was calculated by Wilcoxon rank sum test*.

*M, male; F, female; FL, Left; R, Right*.

### Assessment of Consistency Between Raters

The value of ICC in this study ranged from 0.70 to 0.83, which indicates a good consistency of rating between raters. The detailed information is shown in Table 2.

**Table 2:** Assessment of consistency of rating between raters.

**visual rating scales**	**position**	**ICC**
MTA	Left	0.83
	Right	0.82
PA	Left	0.74
	Right	0.74
AT	Left	0.74
	Right	0.75
OF	Left	0.70
	Right	0.70
AC	Left	0.71
	Right	0.70
FI	Left	0.77
	Right	0.75

### Correlation Analysis Between Each Visual Rating Scale and MMSE

All six visual rating scales have a negative correlation with scores of the MMSE. The correlation coefficient range was −0.35 ~ −0.48 and was statistically significant (*p* < 0.05). The detailed information is shown in Table 3.

**Table 3:** Correlation analysis between each visual rating scale and MMSE.

**Visual rating scales**	**Correlation coefficient**	***p*^**a**^**
MTA	−0.41	< 0.01
PA	−0.37	< 0.01
AT	−0.42	< 0.01
OF	−0.35	< 0.01
AC	−0.48	< 0.01
FI	−0.40	< 0.01

a *After correction of age and gender*.

### VBM and Correlation Analysis Between the Score of Each Visual Rating Scale and the Volume of Gray Matter of Corresponding Brain Regions

In total, 89 individuals, including 42 AD cases and 47 normal controls, were enrolled to perform the analysis of VBM. The overview of the participants' clinical data is described in Table 4. Research results indicated that there was a significant negative correlation (*p* < 0.05) between each visual rating scale and the volume of gray matter of the corresponding brain regions. Detailed information is described in Table 5.

**Table 4:** Demographic and clinical data of the AD cases and normal controls.

	**AD cases**	**Normal controls**	***p*-value**
Number	42	47	
Age	59.38 ± 8.70	57.21 ± 8.37	0.234^a^
Sex(M/F)	17/25	14/33	0.374^b^
Education (years)	9.80 ± 2.68	9.08 ± 2.82	0.848^a^
MMSE	16.87 ± 6.33	27.22 ± 1.30	-
CDR	1.02 ± 0.50	-	-
ADL	27.80 ± 7.41	-	-
NPI	8.02 ± 9.60	-	-

a *p-value was calculated by student's test*.

b *-value was calculated by χ^2^-tests*.

*M, male; F, female*.

**Table 5:** Correlation analysis between each visual rating scale and the volume of gray matter of corresponding brain regions.

**Corresponding brain region vs. visual rating scale**	**Correlation coefficient**	***p*-value^**a**^**
MTA	−0.50	< 0.01
PA	−0.45	< 0.01
AT	−0.22	0.0042
OF	−0.30	< 0.01
AC	−0.31	< 0.01
FI	−0.42	< 0.01

a *After correction of age, gender and intracranial volume*.

### The Value of a Single Visual Rating Scale in the Diagnosis of Mild and Moderate-Severe AD

The patients with AD were divided into two groups (mild AD, moderate-severe AD) according to the MMSE scores. Mild AD was defined as follows: 18 points ≤ MMSE ≤ 23points (19). The age- and gender-matched cognitively normal subjects were selected randomly as the controls. The concrete clinical data are described in Tables 6, 7.

**Table 6:** Clinical data of the mild AD cases and normal controls.

	**AD Cases**	**Normal controls**	***p*-value**
Number	43	43	
Age (years)	63.48 ± 10.93	63.00 ± 9.80	0.819^a^
Sex(M/F)	11/32	14/29	0.635^b^
MMSE	21.12 ± 2.78	27.52 ± 1.30	-
CDR	0.75 ± 0.35	-	-
ADL	22.56 ± 2.93	-	-
NPI	7.88 ± 9.50	-	-

a *p-value was calculated by student's test*.

b *p-value was calculated by χ^2^-tests*.

*M, male; F, female*.

**Table 7:** Clinical data of the moderate and severe AD cases and normal controls.

	**AD cases**	**Normal controls**	***p*-value**
Number	57	57	
Age (years)	63.80 ± 7.20	63.42 ± 9.29	0.80^a^
Sex (M/F)	20/37	21/36	1.00^b^
MMSE	11.60 ± 4.32	27.47 ± 1.32	-
CDR	2.00 ± 0.60	-	-
ADL	32.24 ± 8.81	-	-
NPI	12.96 ± 14.51	-	-

a *p-value was calculated by student's test*.

b *p-value was calculated by χ^2^-tests*.

*M, male; F, female*.

The optimal cutoff of all six visual rating scales which could distinguish moderate-severe AD cases from normal controls was 1.5. The sensitivity, specificity, and AUC of all six visual rating scale ranges were 0.51–0.72, 0.56–0.97, and 0.68–0.80, respectively. Among them, the AUC of MTA and OF ranked the highest and were both 0.80. The detailed data are described in Table 8.

**Table 8:** The AUC, sensitivity and specificity of single visual rating scale in the mild AD cases.

**Visual rating scale**	**position**	**Cutoff**	**sensitivity**	**specificity**	**AUC**
MTA	L	1.5	0.63	0.95	0.79
	R	1.5	0.67	0.90	0.80
	M	1.5	0.62	0.95	0.79
PA	L	1.5	0.72	0.56	0.69
	R	1.5	0.72	0.56	0.69
	M	1.5	0.72	0.56	0.69
AT	L	1.5	0.65	0.66	0.68
	R	1.5	0.67	0.86	0.77
	M	1.5	0.65	0.86	0.77
OF	L	1.5	0.65	0.97	0.80
	R	1.5	0.62	0.97	0.79
	M	1.5	0.63	0.96	0.79
AC	L	1.5	0.51	0.95	0.75
	R	1.5	0.51	0.95	0.76
	M	1.5	0.51	0.95	0.76
FI	L	1.5	0.62	0.73	0.70
	R	1.5	0.62	0.73	0.70
	M	1.5	0.60	0.75	0.70

*L, left; R, right; M, mean*.

The optimal cutoff of all six visual rating scales, which could distinguish moderate-severe AD cases from normal controls was 1.5. The sensitivity, specificity, and AUC of all the six visual rating scale ranges were 0.78–0.87, 0.68–0.95, and 0.77–0.90, respectively. Among them, the AUC of MTA, AC, and OF ranked the highest and were all 0.90. The detailed data are described in Table 9.

**Table 9:** The AUC, sensitivity and specificity of single visual rating scale in the moderate and severe AD cases.

**Visual rating scale**	**Position**	**Cutoff**	**Sensitivity**	**Specificity**	**AUC**
MTA	L	1.5	0.86	0.86	0.89
	R	1.5	0.87	0.85	0.90
	M	1.5	0.86	0.89	0.90
PA	L	1.5	0.86	0.74	0.85
	R	1.5	0.86	0.74	0.85
	M	1.5	0.86	0.74	0.85
AT	L	1.5	0.84	0.80	0.86
	R	1.5	0.82	0.85	0.86
	M	1.5	0.82	0.91	0.89
OF	L	1.5	0.82	0.95	0.90
	R	1.5	0.80	0.93	0.89
	M	1.5	0.82	0.94	0.90
AC	L	1.5	0.79	0.95	0.90
	R	1.5	0.78	0.94	0.90
	M	1.5	0.80	0.95	0.90
FI	L	1.5	0.84	0.68	0.77
	R	1.5	0.83	0.68	0.77
	M	1.5	0.84	0.84	0.88

*L, left; R, right; M, mean*.

### The Value of a Prediction Model Combining Multiple Visual Rating Scales in the Diagnosis of Mild AD

Through the analysis of binary logistic regressions, three concomitant variables were enrolled, including two visual rating scales (MTA, OF) and age. The diagnostic prediction model was established as follows: Score = B_MTA(score)_ + B_OF(score)_ −1.58 (age < 65 years); Score = B_MTA(score)_ + B_OF(score)_ −4.09 (age ≥65 years). When the value of the model ≥0, the person is estimated to have AD, when the value of the model < 0, the person is estimated to be cognitively normal. The concrete parameters are described in detail in Tables 10, 11. The sensitivity, specificity, and AUC of this model in distinguishing mild AD cases from normal controls were 0.74, 0.93, and 0.92, respectively. Compared to the most effective single visual rating scale, MTA (AUC: 0.79, sensitivity: 0.62, specificity: 0.95), the difference between them was statistically significant (*p* < 0.05).

**Table 10:** Setting list of dummy variable parameters.

		**Dummy variable parameters**			
		**(1)**	**(2)**	**(3)**	**(4)**
MTA	0	0	0	0	0
	1	1	0	0	0
	2	0	1	0	0
	3	0	0	1	0
	4	0	0	0	1
OF	0	0	0	0	
	1	1	0	0	
	2	0	1	0	
	3	0	0	1	
Age	≥65	1			
	< 65	0			

**Table 11:** Variables and related coefficient of regression equation.

	**B**	**OR**
MTA(0)		
MTA(1)	0.78	2.2
MTA(2)	4.31	74.56
MTA(3)	21.08	3.50 × 10^9^
MTA(4)	20.06	5.13 × 10^8^
OF(0)		
OF(1)	0.17	1.19
OF(2)	1.78	5.94
OF(3)	22.02	3.65 × 10^9^
Age (≥65 years)	−2.51	0.82
Constant	−1.58	

*B, regression coefficient; OR, odds ratio*.

## Discussion

As one of the common screening means, structural MRI of the brain plays a key role in the diagnosis and differential diagnosis of dementia (20–22). Visual rating scales based on structural MRI could increase the accuracy of imaging assessment and provide radiologists and other clinical researchers a framework to describe the structural image.

Evaluation of the visual rating scales was inevitably subjective; however, two studies (23, 24) indicated that two trained radiologists could have good consistency. Our study showed similar results. Consequently, we can conclude that the visual rating scales have a good repeatability and can be applied to clinical practice.

One of the most widely used screening tools for AD, the MMSE, was established by Folstein in 1975 (25). The MMSE covers multiple cognitive domains, including orientation, memory, attention and calculation power, executive functioning, language and visuospatial functioning. The results of our study showed that all six visual rating scales had a negative correlation with the MMSE score. Our findings align with the fact that the MMSE is a comprehensive rating scale covering multiple cognitive domains and the atrophy of corresponding brain regions can decrease the points of corresponding cognitive domains. A previous study indicated that MTA and PA were negatively correlated with MMSE scores independently in AD cases (9), which was consistent with our study.

The visual rating scale of MTA was first put forward by Scheltens et al., and the sensitivity and specificity of MTA in distinguishing AD from normal controls were 81 and 67%, respectively (7). In our study, the sensitivity and specificity of MTA were (62%, 95%) in mild AD cases and (86%, 89%) in moderate and severe AD cases. The subtle difference between the two studies may be due to the stratification of AD according to severity of disease. The previous study indicated that the sensitivity and specificity of FI in distinguishing early-onset AD from normal controls were 74 and 94%, respectively (15), close to our results (84 and 84%). AT was mainly used to estimate the atrophy of the temporal lobe in frontotemporal dementia (FTD) cases. A previous study indicated that the scores of AT related to the extent of atrophy at autopsy (11). However, AT could effectively differentiate AD from normal controls in our study, indicating that atrophy of the anterior temporal lobe can be found in AD cases. Anterior cingulate plays a key role in execution function. One study indicated that the volume of the anterior cingulate cortex had a negative correlation with two items of execution function in amnestic mild cognitive impairment (aMCI) cases, and it decreased significantly compared with normal controls (26). The significant difference of AC between AD cases and normal controls in our study was similar to the results of the preceding study. The visual rating scale, PA, focusing on the posterior cingulate, precuneus, parieto-occipital sulcus, and parietal cortex, was put forward by Koedam et al. (9). To date, as the only visual rating scale targeting the posterior portion of the brain, PA can be used to differentiate AD from normal controls and other dementias. The sensitivity and specificity of PA in distinguishing AD from normal controls were 58 and 95%, respectively (9), which was superior to the performance in mild AD cases and inferior to the performance in moderate and severe AD cases in our study. Atrophy of multiple brain regions, including the insula lobe, anterior hippocampus, temporal pole, and orbital-frontal cortex, was found in AD cases compared to normal controls (12). The discovery indicates that there may be diffuse atrophy in AD brains, which is consistent with the high sensitivity and specificity of each single visual rating scale in distinguishing moderate and severe AD from normal controls in our study.

In our study, six visual rating scales and age were enrolled in the logistic regression equation to explore a prediction model that could distinguish mild AD cases from normal controls. The ultimate prediction model included three variables: MTA, OF, and age stratification. The sensitivity and specificity of this model in distinguishing mild AD cases from normal controls were 0.74 and 0.93, respectively, superior to the most effective single visual rating scale, MTA (sensitivity: 0.62, specificity: 0.95), and the difference between them was statistically significant (*p* < 0.05). To date, many studies confirm that medial temporal atrophy is related to AD (27, 28). MTA was verified as practical and repeatable in clinical practice, and was related to the volume of the hippocampus (29, 30). As well, increasing evidence indicated that the orbital frontal cortex was involved in the early stage of AD (31, 32). These studies presented corresponding morphological changes of the medial temporal lobe and orbital frontal lobe cortex in the early stage of AD. Therefore, the combined use of MTA and OF may be superior to a single visual rating scale in the early diagnosis of AD and the higher the score of the model, the greater the possibility of AD.

Age is one of the influencing factors in brain atrophy, and visual rating scales after age stratification would have better accuracy (15, 33, 34). A previous study indicated that the score of MTA in the normal controls under the age of 70 years ranged from 0 to 1, and the score of MTA in the normal controls at the age of 70–80 years ranged from 0 to 2 (35). Given that there was research taking 65 years old as the cutoff point in the process of combining six visual rating scales to diagnose AD (15), our study divided AD cases into two groups (early-onset AD and late-onset AD) and enrolled age as a variate into the research model to be selected. The results showed that age stratification was enrolled into the prediction model finally, and the regression coefficient of age stratification was negative, which indicated that brain atrophy to some extent in the aged cases may be normal senile atrophy.

Our study found that every single visual rating scale could effectively distinguish AD cases from normal controls and was repeatable in the Chinese population, especially for moderate-severe AD cases. For mild AD cases, the prediction model of combining MTA, OF, and age stratification was better than using a single visual rating scale. There are still some limitations in our study as follows: The sample size was relatively small and the enrolled cases were mainly clinically probable typical AD cases. Consequently, a large sample size, detailed age stratification, and enrolling more AD cases, supported by positron emission tomography-computed tomography (PET-CT), CSF biomarkers, and autopsy, are necessary to obtain more accurate discoveries.

## Ethics Statement

All procedures performed in studies involving human participants were approved by the Ethics Committee of Xiangya Hospital, Central South University in China, which was in accordance with ethical standards of the institutional and/or national research committee and with the 1964 Helsinki declaration and its later amendments or comparable ethical standards. Written informed consent was obtained from all subjects.

## Author Contributions

LS and TX involved in the study design. ZY and CP were responsible for the enrollment of the participants. ZY, CP, ML, and WZ were responsible for the estimation of the visual rating scales. LS, BT, and XY were responsible for the confirmation of the participants. ZY wrote the manuscript. LS and BJ modified and revised the manuscript. All authors have read and approved the final version of the manuscript.

### Conflict of Interest Statement

The authors declare that the research was conducted in the absence of any commercial or financial relationships that could be construed as a potential conflict of interest.
